# Methamphetamine-Fentanyl Polysubstance Administration Produces Social Deficits and Corticolimbic Stress-Reward Circuit Adaptations

**DOI:** 10.21203/rs.3.rs-9306429/v1

**Published:** 2026-04-23

**Authors:** Leah M. Salinsky, Joshua L. Fox, Kyra C. Diaz, Bradley C. Timmons, Susan M. Ferguson

**Affiliations:** University of Washington

**Keywords:** Methamphetamine, Fentanyl, Polysubstance use, Psilocybin, Social preference, CRHR1, OPRM1, Medial prefrontal cortex, Nucleus accumbens, Addiction

## Abstract

**Rationale and Objectives:**

Polysubstance use involving psychostimulants and opioids is increasingly prevalent and associated with elevated overdose risk, relapse vulnerability, and poor treatment outcomes. However, the neurobehavioral consequences of opioid-stimulant use remain poorly understood. We evaluated whether repeated methamphetamine-fentanyl polysubstance treatment disrupts social preference during withdrawal, whether psilocybin treatment restores sociability, and whether these manipulations alter corticolimbic expression of stress- and opioid-related genes.

**Methods:**

Male and female rats received injections of methamphetamine and fentanyl or saline and were assessed for social preference at baseline and following 10 days of withdrawal. On withdrawal day 10, rats received psilocybin or saline and were reassessed for sociability 24 h later. *CRHR1* and *OPRM1* expression in the medial prefrontal cortex (mPFC) and nucleus accumbens (NAc) were quantified by RT-qPCR.

**Results:**

Withdrawal from 14-days of polysubstance treatment reduced social preference, replicating prior findings of withdrawal-associated social dysfunction. Psilocybin pretreatment did not restore social preference at the time point examined. In the mPFC, psilocybin bidirectionally altered *CRHR1* expression depending on drug history, decreasing expression in saline-treated controls, while increasing expression following polysubstance treatment. In the NAc, polysubstance administration reduced *CRHR1* expression. *OPRM1* expression showed sex-dependent regulation with a marked reduction in the NAc of females following polysubstance treatment and evidence of sex-dependent effects in the mPFC.

**Conclusions:**

Methamphetamine-fentanyl treatment produces persistent social deficits during withdrawal and region- and sex-dependent corticolimbic transcriptional adaptations *in vivo*. Although psilocybin did not restore sociability, it produced drug history-dependent regulation of cortical *CRHR1*.

## Introduction

Polysubstance use involving psychostimulants and opioids has become a defining feature of the current overdose crisis, with methamphetamine and fentanyl co-use associated with elevated morbidity and mortality, relapse vulnerability, and limited treatment efficacy^1-4^. While substantial work has characterized the reinforcing properties of individual drugs, far less is known about the neurobehavioral consequences of opioid-stimulant polysubstance use.

Beyond drug intake itself, substance use disorders are accompanied by profound disruptions in socioaffective functioning^5-10^. Social withdrawal and reduced social motivation are increasingly recognized as clinically relevant factors in on-going substance use, contributing to negative emotional states and relapse risk^11,12^. Preclinical models of social functioning provide an opportunity to quantify these impairments using translational measures of sociability and social preference. In recent work, we demonstrated that repeated administration of methamphetamine and fentanyl induces a robust deficit in social interaction, an effect not observed following administration of either drug alone^13^. The present study replicates this polysubstance withdrawal-associated social deficit in an independent cohort, strengthening the behavioral phenotype and supporting its utility as a translational endpoint for polysubstance-induced dysfunction. Notably, these results suggest that opioid-stimulant exposure is detrimental to social functioning in rodent models of addiction, consistent with the heightened psychiatric burden observed in individuals who co-use stimulants and opioids.

Classic serotonergic psychedelics have re-emerged as promising candidates for the treatment of substance use disorders, with accumulating evidence that serotonin (5-HT) 2A receptor (5-HT_2A_R) agonists can reduce drug-seeking and modulate affective and motivational processes^11,14-17^. In preclinical studies, psychedelic compounds have been shown to attenuate withdrawal-related behavioral disturbances^18,19^. Consistent with this, we found that administering the psychedelic (−)-2,5-dimethoxy-4-iodoamphetamine (DOI) shortly before testing reversed opioid-stimulant-induced reductions in sociability^13^. However, while DOI has been widely characterized in preclinical studies^20^, formal studies of DOI in clinical populations have not been conducted. Whether these effects generalize to psilocybin, a psychedelic with distinct pharmacological properties and growing clinical relevance is unknown.

Psilocybin can engage neuroplasticity-related mechanisms within cortical circuits, including the medial prefrontal cortex (mPFC), over extended post-administration intervals^21-23^. These sustained adaptations may be particularly relevant for withdrawal-associated negative affect and socioaffective dysfunction as chronic drug exposure is known to remodel stress- and reward-related signaling across corticolimbic circuitry^24-29^. In particular, the mPFC and the nucleus accumbens (NAc) form a central circuit regulating social motivation, affective state, and relapse vulnerability^30-35^. Prefrontal cortex projections to the NAc contribute to the integration of emotional and motivational information, and dysregulation within this pathway has been implicated in both addiction-related behavioral responses and social dysfunction^30,35,36^.

The NAc is a key node for reward processing, encoding the reinforcing value for both drug-related and social stimuli^37,38^. Social interaction itself is a potent natural reward, and accumbal signaling is critical for mediating social approach behavior and social motivation^32,39^. Conversely, chronic drug exposure and abstinence can shift reward circuitry toward negative reinforcement states, in which stress and dysphoria become dominant drivers of behavior^35^. Thus, transcriptional adaptations within the mPFC-NAc circuit may represent an important mechanistic link between polysubstance withdrawal and persistent social deficits.

Stress neuropeptide systems within this circuit are strongly engaged during drug withdrawal^40^. Corticotropin-releasing hormone signaling through the corticotropin-releasing hormone receptor 1 (CRHR1) plays a central role in coordinating behavioral and endocrine responses to stress and has been implicated in withdrawal-associated negative affect, anxiety-like behaviors and relapse-related drug seeking^41-45^. Thus, CRHR1 signaling within corticolimbic circuitry may therefore contribute not only to heightened stress responsivity during withdrawal, but also to disruptions in social motivation.

In parallel, μ-opioid receptor (OPRM1/MOR) signaling is critically involved in reward regulation and social function^39,46^. Endogenous opioid systems contribute to the hedonic and motivational components of social interaction, and MOR activation within mesocorticolimbic regions is thought to facilitate social reward and affective behaviors^39,47^. Given the established interactions between opioid and dopaminergic pathways^48^, polysubstance exposure may produce unique adaptations in MOR-related transcription that alter social reinforcement processes during abstinence.

Although serotonergic psychedelics are increasingly recognized for their ability to modulate affective and motivational circuitry^49^, the extent to which psilocybin alters CRH- and opioid-related transcriptional pathways following opioid-stimulant polysubstance use is not yet understood. Characterizing *CRHR1* and *OPRM1* gene regulation within the mPFC and NAc may therefore provide insight into the molecular substrates underlying persistent social dysfunction during drug withdrawal and the circuit-level mechanisms through which psychedelics could exert their therapeutic effects.

We investigated whether psilocybin pretreatment administered 24 hours prior to behavioral assessment alters withdrawal-associated social deficits following repeated methamphetamine-fentanyl treatment in male and female rats. Using a longitudinal social preference design, sociability was assessed at baseline and again following withdrawal. In addition, we quantified *CRHR1* and *OPRM1* mRNA expression in the mPFC and NAc to identify stress- and opioid-related transcriptional adaptations associated with polysubstance drug withdrawal and psychedelic treatment. We hypothesized that psilocybin pretreatment would reverse withdrawal-induced reductions in sociability and that polysubstance and psilocybin treatment would modulate stress- and reward-related molecular signatures within corticolimbic circuitry.

## Materials and Methods

### Animals.

**Figure 1:**
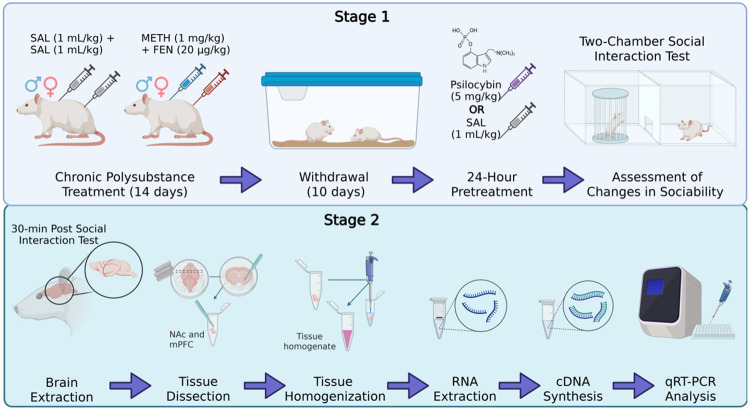
Experimental design to evaluate psilocybin effects on polysubstance withdrawal–induced social deficits and associated gene expression changes. **Stage 1:**Male and female rats received treatment for 14 days with either saline (SAL; 1 mL/kg) or methamphetamine (METH; 1 mg/kg) plus fentanyl (FEN; 20 μg/kg). Following 10 days of withdrawal, animals were administered psilocybin (5 mg/kg) or SAL (1 mL/kg) 24 hours prior to behavioral testing. Sociability was assessed using a two-chamber social interaction test, and changes in social interaction were quantified relative to baseline. **Stage 2:** Thirty minutes following behavioral testing, brains were collected for molecular analysis. The nucleus accumbens (NAc) and medial prefrontal cortex (mPFC) were dissected, homogenized, and processed for RNA extraction. cDNA was synthesized and gene expression was quantified using quantitative real-time PCR (qRT-PCR).

Male Sprague–Dawley rats (n = 20; Inotiv Inc., Livermore, CA, USA) weighing 225–250 g upon arrival and female Sprague-Dawley rats (n = 20; Inotiv Inc., Livermore, CA, USA) weighing 180–200 g upon arrival were allowed to acclimate for seven days in a colony room with controlled temperature and humidity on a 12-h light/dark cycle (lights on 0600–1800 h). Rats were pair-housed according to sex and weighed and handled daily for at least five minutes by experimenters throughout the study. Food (LabDiet, Irradiated PicoLab) and water were available ad libitum throughout all phases of the studies except during behavior sessions. Behavioral experiments took place during the light phase of the light/dark cycle and all experiments were carried out in accordance with the *National Research Council Guide for the Care and Use of Laboratory Animals* and with approval from the University of Washington Institutional Animal Care and Use Committee. The overall experimental design is illustrated in Fig. 1.

### Drugs.

Fentanyl HCl (National Institute on Drug Abuse, Bethesda, MD) administered subcutaneously (*sc*), rac-Methamphetamine HCl (National Institute on Drug Abuse, Bethesda, MD) administered intraperitoneally (*ip*), and psilocybin (National Institute on Drug Abuse, Bethesda, MD) administered *sc* were all dissolved in 0.9% NaCl and administered at a volume of 1 ml/kg.

### Two-Chamber Side-Bias Pretest and Counterbalancing.

Following acclimation to the colony, subject rats were transferred to the experimental room and allowed to acclimate to room conditions for 15 min prior to manipulation. To account for innate side preferences, each subject rat underwent a 20-min pretest in the empty apparatus, during which it was allowed to freely explore both compartments. Time spent in each compartment was quantified to determine baseline side preference. Animals were then counterbalanced such that assignment of the social stimulus rat versus the empty compartment was balanced across subjects and did not systematically align with baseline side bias.

### Baseline Sociability Evaluation (Two-Chamber Social Preference Task).

Rats were assessed for social preference using a two-chamber social interaction task. All behavioral tests were conducted in the same order across animals to minimize variability across testing sessions. The apparatus consisted of a plexiglass rectangular box divided into two equal compartments with free access between compartments (36 cm × 36 cm × 29 cm). Subject and stimulus rats were transferred to the experimental room and allowed to acclimate to room conditions for 15 min prior to experimentation. Prior to testing, subject rats were allowed to freely explore the apparatus for 5 min (habituation period). This free exploration period was repeated prior to all subsequent social interaction tests. During the sociability test, a strain-, age-, and sex-matched novel stimulus rat was placed under a rectangular perforated plastic crate (24.4 cm × 16.5 cm × 9.7 cm) in one compartment, while the opposite compartment remained empty. At the start of the trial, the subject rat was placed in the apparatus with free access to both compartments for 10 min. Social preference was quantified as the time spent in the compartment containing the social stimulus (TS) relative to time spent in the empty compartment (TE). A social preference index was calculated as:

$$Social\;Index=\frac{T_{S}-T_{E}}{T_{S}+T_{E}}$$

Between subjects, stimulus location (left vs right compartment) was counterbalanced based on the pretest to control for innate side preference.

### Tracking Using DeepLabCut.

The location of subject rats within the social interaction apparatus was automatically tracked using the ‘single animal’ class in DeepLabCut (DLC) software (v2.3.10)^50^. A total of 193 training frames were automatically extracted using the k-means algorithm. A training dataset was generated by manually labeling four body points (nose, left ear, right ear, tail base) using DLC’s *napari* plug-in (v0.4.18). The resulting tracking data (CSV format), representing body-part coordinates across the session, were exported for downstream behavioral quantification.

### Region of Interest Evaluation Using SimBA.

Time spent in each compartment was quantified using the Simple Behavioral Analysis (SimBA) toolkit (v2.8)^51^. Experimental videos and corresponding DLC tracking outputs were imported into a SimBA project and analyzed using the ROI interface. ROIs were defined for each video to correspond to the social stimulus compartment and the empty compartment. ROI outputs were used to quantify time spent in each compartment, which served as the primary measure of sociability and was used to calculate the social preference index.

### Administration of Chronic Drug Treatment.

Rats received 14 daily treatments of either saline + saline or methamphetamine + fentanyl with one injection in the morning (AM, 10:00–11:00 h) and one in the afternoon (PM, 14:00–15:00 h). Injections were spaced 4 hours apart so that testing for both drugs occurred during the same phase of the light/dark cycle. Methamphetamine (1 mg/kg, *ip*) was administered in the morning, and fentanyl (20 ug/kg, *sc*) was administered in the afternoon. The control group received two injections of saline (1 mL/kg, *ip*), one in the morning and one in the afternoon. This dose regimen was chosen based on our previous findings demonstrating a distinct polysubstance-associated social deficit in male and female Sprague Dawley rats that was not present in single-substance treated rats^13^.

### Assessment of social interaction following drug withdrawal.

Following completion of the drug treatment regimen, all subjects were returned to their home cages for 10 days of withdrawal. On withdrawal day 10, rats received an injection of either saline (1 mL/kg) or psilocybin (5 mg/kg) and were returned to their home cages. Twenty-four hours later, all subjects were reassessed for sociability using the two-chamber protocol described above. This dose of psilocybin was utilized as it has been shown to induce neuroplasticity within the corticolimbic system and we chose a 24-hour time point due to the growing abundance of evidence displaying behavioral adaptations 24-hours following treatment with a neuroplastic dose of psilocybin^21-23^.

### Tissue Collection.

Thirty minutes following completion of the sociability assessment, rats were briefly anesthetized via CO_2_ inhalation and decapitated. Brains were rapidly extracted and placed on ice. Tissue dissection was performed using a coronal rodent brain matrix, and 2-mm sections were obtained. The medial prefrontal cortex (mPFC) and nucleus accumbens (NAc) were microdissected over ice, flash frozen on powdered dry ice, and stored at −80°C until RNA isolation.

### Tissue Homogenization and RNA Extraction.

Brain regions were homogenized in TRIzol reagent (Cat No: 15-596-026), and RNA was extracted according to the manufacturer’s instructions. RNA concentration, A260/A280, and A260/A230 ratios were determined by Nanodrop 1000 spectrophotometer (Thermo Fisher Scientific, Delaware, USA).

### Complementary DNA Synthesis.

Before complementary DNA (cDNA) synthesis, the RNA concentrations of all samples were adjusted to 200 ng/*μ*L for both PFC and NAc. RNA was reversed transcribed using the Invitrogen SuperScript III First-Strand System (Cat No: 18-080-051) following the manufacturer’s instructions. The cDNA samples were stored at −20°C until real-time quantitative polymerase chain reaction (qRT-PCR) analysis.

### Real-time qPCR.

qRT-PCR was conducted using TaqMan^™^ Fast Advanced Master Mix (Applied Biosystems; Cat No: 4444557) and commercially available probes for Cyclophilin A (*PPIA*; Cat ID: Rn00690933_m1), *OPRM1* (Cat ID: Rn01430371_m1), and *CRHR1* (Cat ID: Rn00578611_m1). Reactions (20 μL) were run at 50°C for 2 min, 95°C for 20 s, followed by 40 cycles of 95°C for 1 s and 60°C for 20 s using the QuantStudio 3 system. Threshold cycle (Ct) values were automatically determined using the QuantStudio analysis software with default threshold settings.

### RNA Quantification and Relative Expression Analysis.

Target gene expression was normalized to the housekeeping gene peptidylprolyl isomerase A (PPIA) using the comparative ΔΔCt method. All samples were run in triplicate with the average Ct utilized for further analysis. For each sample, ΔCt values were calculated as: ΔCT=CT(geneofinterest)−CT(PPIA) Relative expression was then quantified using the ΔΔCt method. To account for potential baseline sex differences in gene expression, ΔΔCt values were calculated relative to the mean ΔCt of sex-matched saline control animals (rats receiving both chronic saline treatment and saline pretreatment): ΔΔCT=ΔCT(Sample)−ΔCT(ControlAvergae. Fold-change values were calculated as: $Fold\;Change=2^{-\Delta \;\Delta \;CT}$ Fold-change values were subsequently log_2_-transformed for statistical analysis. Log_2_ fold-change values were used for all downstream statistical analyses.

### Statistical Analysis of Social Index.

Social interaction behavior was quantified using a social index measured at two timepoints within the same animals: baseline (T1), prior to any treatment, and post-withdrawal (T2), following 14 days of saline (SAL) or fentanyl + methamphetamine (POLY) administration, 10 days of withdrawal, and saline or psilocybin administered 24 h prior to T2 testing.

Data were analyzed using a linear mixed-effects model with Time (T1 vs T2) as a within-subject factor and Chronic Treatment, Pretreatment, and Sex as between-subject factors. Subject was included as a random intercept. The full model included all interaction terms:

SocialIndex∼Time×ChronicTreatment×Pretreatment×Sex

Planned contrasts were performed on model-based change scores (Δ = T2 - T1). Multiple comparisons were corrected using the Holm–Šídák procedure. Analyses were conducted in Python using the statsmodels package. Model parameters were estimated via maximum likelihood with statistical significance defined as α = 0.05.

### Statistical Analysis of Gene Targets.

For *OPRM1* and *CRHR1*, relative expression was quantified using the comparative ΔΔCt method after normalization to the housekeeping gene PPIA. To minimize the influence of baseline sex differences in expression, ΔΔCt values for each subject were calculated relative to the mean ΔCt of the sex-matched saline control group (i.e., rats receiving both chronic saline treatment and saline pretreatment within the same sex). Fold-change values were then log2 transformed for statistical analysis.

Log_2_ fold-change values were analyzed using a three-way Gaussian generalized linear model with Sex (male vs female), Chronic Treatment (saline vs polysubstance), and Pretreatment (saline vs psilocybin) as between-subject factors:

GeneExpression(foldchange)∼Sex×ChronicTreatment×Pretreatment

Type III sums of squares were used to evaluate main effects and interaction terms. Statistical significance was defined as α = 0.05. When significant interaction effects were detected, follow-up analyses were performed using planned simple-effects comparisons based on estimated marginal means from the fitted model. Multiple comparisons were corrected using the Holm–Šídák procedure. All analyses were conducted in Python using the statsmodels package.

## Results

### Sociability following polysubstance exposure.

Social interaction was quantified using a social preference index measured at baseline (T1), prior to drug treatment, and again following withdrawal (T2), assessed on withdrawal day 11, 24 h after saline or psilocybin pretreatment. Social index data were analyzed using a linear mixed-effects model with Time (T1 vs T2) as a within-subject factor and Chronic Treatment (saline vs POLY), Pretreatment (saline vs psilocybin), and Sex (male vs female) as between-subject factors, with Subject included as a random intercept.

**Figure 2:**
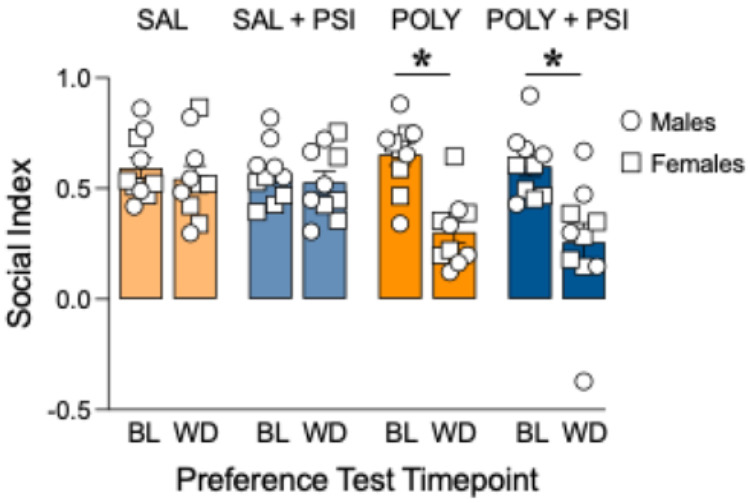
Polysubstance administration induces persistent social deficits during withdrawal that were not prevented by psilocybin pretreatment. Rats underwent baseline (BL) social testing prior to 14 days of saline (SAL) or methamphetamine + fentanyl (POLY) treatment, followed by 10 days of withdrawal (WD). Psilocybin (PSI; 5 mg/kg) or SAL (1 mL/kg) was administered 24 hours prior to the second social test. Social index (mean ± SEM) was reduced in the POLY groups after withdrawal relative to baseline (*p < 0.05). Psilocybin pretreatment did not restore social interaction in the POLY group. Individual data points represent males (circles) and females (squares). n=5 per sex/group.

Across all groups, social index significantly declined from baseline to the post-withdrawal assessment (Time effect: β = −0.433, z = −5.81, *p* < 0.0001, Fig. 2). While there were no significant main effects of Chronic Treatment, Pretreatment, or Sex on overall social index levels (all *p* > 0.12, Fig. 2), a significant Time × Chronic Treatment interaction was observed (β = 0.306, z = 2.90, *p* = 0.004, Fig. 2), indicating that the magnitude of behavioral change across time differed between the saline and POLY groups. In contrast, psilocybin pretreatment did not significantly influence social index trajectories (Time × Pretreatment: *p* = 0.94, Fig. 2), and no higher-order interactions involving Pretreatment or Sex reached significance (all *p* > 0.66, Fig. 2).

Planned comparisons of model-based change scores (Δ = T2 - T1) demonstrated that repeated POLY treatment produced a robust reduction in sociability relative to saline controls (Δ difference = −0.304, Holm–Šídák adjusted *p* < 0.0001, Fig. 2). However, psilocybin pretreatment did not significantly alter social index change scores within POLY animals (adjusted *p* = 0.99, Fig. 2) or saline-treated controls (adjusted *p* = 0.99, Fig. 2). Together, these findings indicate that repeated administration of methamphetamine + fentanyl induces persistent social deficits during withdrawal, and that acute psilocybin pretreatment does not restore sociability at the assessed 24-hour timepoint.

### Gene Expression Analysis of OPRM1 Alterations in the Medial Prefrontal Cortex and Nucleus Accumbens

#### Medial Prefrontal Cortex.

**Figure 3:**
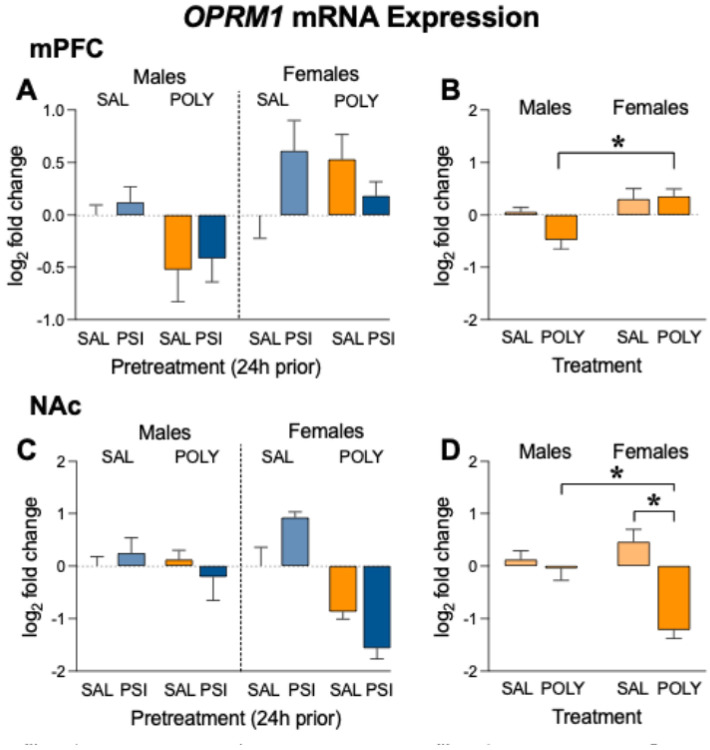
*OPRM1* (μ-opioid receptor) mRNA expression in corticolimbic regions following polysubstance and psilocybin treatment. (A) *OPRM1* expression in the mPFC displayed for all experimental groups (Sex × Chronic Treatment × Pretreatment). (B) mPFC *OPRM1* expression collapsed across pretreatment to show the significant sex-dependent effects of repeated polysubstance administration. (C) *OPRM1* expression in the NAc displayed for all experimental groups. (D) NAc *OPRM1* expression collapsed across pretreatment to show the significant Sex × Chronic Treatment interaction, revealing a pronounced reduction in *OPRM1* expression in females following polysubstance treatment relative to males. Points represent individual animals and bars represent mean ± SEM. Gene expression values represent log_2_ fold change relative to saline-treated controls within each sex. Statistical analyses were performed using a three-way Gaussian generalized linear model (Sex × Chronic Treatment × Pretreatment) with Type III sums of squares and Holm–Šídák correction for planned comparisons. Collapsed panels are presented for visualization of statistically significant effects identified in the full factorial model. *p < 0.05, n=5 per sex/group.

*OPRM1* mRNA expression in the medial prefrontal cortex (mPFC) was analyzed using a three-way Gaussian generalized linear model with Chronic Treatment (saline vs polysubstance), Pretreatment (saline vs psilocybin), and Sex (male vs female) as between-subject factors. No significant main effects of pretreatment (F_1,32_ = 0.63, *p* = 0.43, Fig. 3A) or chronic treatment (F_1,32_ = 2.41, *p* = 0.13, Fig. 3A) were detected. In contrast, a significant main effect of sex was observed (F_1,32_ = 12.07, *p* = 0.0015, Fig. 3A-B), indicating that OPRM1 expression differed between males and females across experimental conditions.

No significant Pretreatment × Sex interaction was detected (F_1,32_ = 0.003, *p* = 0.95, Fig. 3A), and the Pretreatment × Chronic Treatment interaction was also not significant (F_1,32_ = 2.47, *p* = 0.13, Fig. 3A). A trend-level Sex × Chronic Treatment interaction was observed (F_1,32_ = 3.56, *p* = 0.068, Fig. 3A-B), suggesting that the transcriptional effects of polysubstance exposure may differ between males and females. The three-way interaction between Pretreatment, Sex, and Chronic Treatment was not significant (F_1,32_ = 2.34, *p* = 0.14, Fig. 3A).

Planned simple-effects comparisons collapsed across pretreatment revealed a trend toward reduced *OPRM1* expression in polysubstance-treated males relative to saline-treated controls (Holm–Šídák adjusted *p* = 0.061, Fig. 3B), whereas no treatment effect was detected in females (Holm–Šídák adjusted *p* = 0.81). Consistent with this pattern, females given methamphetamine + fentanyl exhibited significantly higher *OPRM1* expression than males within the same treatment condition (Holm–Šídák adjusted *p* = 0.0025, Fig. 3B). Together, these findings suggest a potential sex-dependent regulation of prefrontal μ-opioid receptor transcription following chronic methamphetamine–fentanyl exposure.

For visualization purposes, panel B displays data collapsed across pretreatment to illustrate the sex-dependent pattern of *OPRM1* expression.

#### Nucleus Accumbens.

*OPRM1* mRNA expression in the nucleus accumbens (NAc) was analyzed using a three-way Gaussian generalized linear model with Sex, Chronic Treatment, and Pretreatment as between-subject factors. A significant main effect of chronic treatment was detected (F_1,32_ = 24.73, *p* = 2.1 × 10^−5^), indicating that polysubstance exposure altered OPRM1 transcription in the NAc relative to saline-treated controls. In addition, a significant main effect of sex was observed (F_1,32_ = 5.08, *p* = 0.031, Fig. 3C-D), suggesting sex-dependent differences in μ-opioid receptor expression.

Importantly, a robust Sex × Chronic Treatment interaction was detected (F_1,32_ = 16.65, *p* = 0.00028), indicating that the transcriptional effects of polysubstance administration differed between males and females (Fig. 3C-D). Planned simple-effects comparisons collapsed across pretreatment revealed that polysubstance exposure produced a pronounced reduction in *OPRM1* expression in females relative to saline-treated controls (Holm–Šídák adjusted *p* < 0.001), whereas no significant treatment effect was detected in males. Consistent with this pattern, females given methamphetamine + fentanyl exhibited significantly lower *OPRM1* expression than males within the same treatment condition (Holm–Šídák adjusted *p* < 0.001).

A significant Pretreatment × Chronic Treatment interaction was also detected (F_1,32_ = 8.80, *p* = 0.0057, Fig. 3C), suggesting that the transcriptional effects of psilocybin pretreatment varied as a function of prior drug history. However, follow-up simple-effects comparisons did not remain significant following correction for multiple comparisons, indicating that these effects were modest. The three-way interaction between Sex, Pretreatment, and Chronic Treatment was not significant (F_1,32_ = 1.97, *p* = 0.17, Fig. 3C).

For visualization purposes, panel D displays data collapsed across pretreatment to illustrate the significant Sex × Chronic Treatment interaction observed in the statistical model.

### Gene Expression Analysis of CRHR1 Alterations in the Medial Prefrontal Cortex and Nucleus Accumbens

#### Medial Prefrontal Cortex.

**Figure 4:**
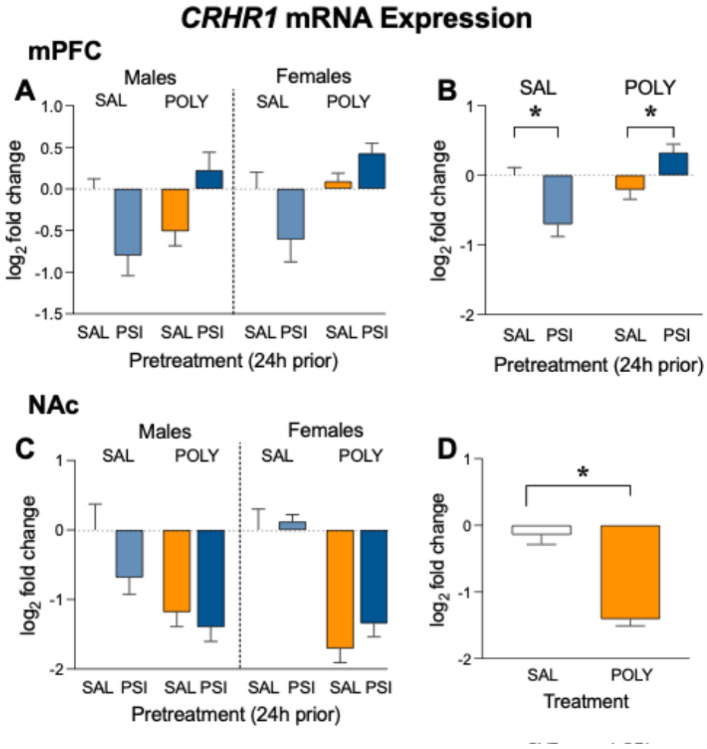
*CRHR1* mRNA expression in corticolimbic regions following polysubstance and psilocybin treatment. (A) *CRHR1* expression in the mPFC displayed for all experimental groups (Sex × Chronic Treatment × Pretreatment). (B) mPFC *CRHR1* expression collapsed across sex to show the significant Pretreatment × Chronic Treatment interaction. Psilocybin pretreatment produced bidirectional regulation of *CRHR1*expression depending on drug history, decreasing *CRHR1* expression in saline-treated controls while increasing *CRHR1* expression following polysubstance administration. (C) *CRHR1* expression in the NAc displayed for all experimental groups. (D) NAc *CRHR1* expression collapsed across sex and pretreatment to show the significant main effect of chronic treatment, demonstrating reduced *CRHR1* expression following polysubstance treatment. Bars represent mean ± SEM. Gene expression values represent log_2_ fold change relative to saline-treated controls within each sex. Statistical analyses were performed using a three-way Gaussian generalized linear model (Sex × Chronic Treatment × Pretreatment) with Type III sums of squares and Holm–Šídák correction for planned comparisons. Collapsed panels are presented for visualization of statistically significant effects identified in the full factorial model. *p < 0.05, n=5 per sex/group.

*CRHR1* mRNA expression in the medial prefrontal cortex (mPFC) was analyzed using a three-way Gaussian generalized linear model with Chronic Treatment (saline vs polysubstance), Pretreatment (saline vs psilocybin), and Sex (male vs female) as between-subject factors. No significant main effect of pretreatment was detected (F_1,32_ = 0.41, *p* = 0.53, Fig. 4A). A trend-level effect of sex was observed (F_1,32_ = 3.42, *p* = 0.074, Fig. 4A), suggesting potential differences in *CRHR1* expression between males and females. In contrast, a significant main effect of chronic treatment was detected (F_1,32_ = 9.43, *p* = 0.0043, Fig. 4A), indicating that methamphetamine + fentanyl treatment altered *CRHR1* transcription in the prefrontal cortex relative to saline-treated controls.

No significant Pretreatment × Sex interaction was detected (F_1,32_ = 0.15, *p* = 0.70, Fig. 4A), and the Sex × Chronic Treatment interaction was not significant (F_1,32_ = 1.31, *p* = 0.26, Fig. 4A). However, a robust Pretreatment × Chronic Treatment interaction was observed (F_1,32_ = 21.27, *p* = 6.1 × 10^−5^, Fig. 4A-B), indicating that the transcriptional effects of psilocybin pretreatment depended strongly on prior polysubstance history. The three-way interaction between Pretreatment, Sex, and Chronic Treatment was not significant (F_1,32_ = 1.18, *p* = 0.28, Fig. 4A), suggesting that this drug-history–dependent effect was not further moderated by sex.

Follow-up simple-effects comparisons collapsed across sex revealed significant but opposing effects of psilocybin depending on chronic drug history. In saline-treated control animals, psilocybin significantly reduced *CRHR1* expression relative to saline (Holm–Šídák adjusted *p* = 0.0016, Fig. 4B). In contrast, in polysubstance-treated animals, psilocybin significantly increased *CRHR1* expression compared with saline (Holm–Šídák adjusted *p* = 0.0084, Fig. 4B). These results indicate a bidirectional and drug-history–dependent modulation of cortical CRH signaling by psilocybin.

For visualization purposes, panel B displays data collapsed across sex to illustrate the significant Pretreatment × Chronic Treatment interaction detected in the statistical model.

#### Nucleus Accumbens.

*CRHR1* mRNA expression in the nucleus accumbens (NAc) was analyzed using the same three-way Gaussian generalized linear model with Chronic Treatment (saline vs polysubstance), Pretreatment (saline vs psilocybin), and Sex (male vs female) as between-subject factors. A significant main effect of chronic treatment was observed (F_1,32_ = 40.79, *p* = 3.56 × 10^−7^, Fig. 4C-D), indicating that chronic methamphetamine +fentanyl administration significantly reduced *CRHR1* expression in the NAc relative to saline-treated controls. A modest main effect of pretreatment was also detected (F_1,32_ = 4.50, *p* = 0.042, Fig. 4C), indicating that psilocybin pretreatment was associated with a small overall change in *CRHR1* expression across animals.

No significant main effect of sex was observed (F_1,32_ = 1.89, *p* = 0.18, Fig. 4C). Additionally, no significant Pretreatment × Chronic Treatment interaction (F_1,32_ = 0.24, *p* = 0.63, Fig. 4C), Sex × Chronic Treatment interaction (F_1,32_ = 0.90, *p* = 0.35, Fig. 4C), or Pretreatment × Sex interaction (F_1,32_ = 0.23, *p* = 0.64, Fig. 4C) was detected. A trend-level three-way interaction between Pretreatment, Sex, and Chronic Treatment was observed (F_1,32_ = 3.43, *p* = 0.073, Fig. 4C), though this effect did not reach statistical significance.

Planned simple-effects comparisons collapsed across pretreatment revealed that polysubstance administration significantly reduced *CRHR1* expression relative to saline-treated controls in both males (Holm–Šídák adjusted *p* = 0.0016, Fig. 4D) and females (Holm–Šídák adjusted *p* = 4.6 × 10^−5^, Fig. 4D). No significant sex differences were observed within either chronic treatment condition. Together, these findings indicate that chronic methamphetamine + fentanyl administration robustly suppresses *CRHR1* expression in the nucleus accumbens independent of sex, while psilocybin pretreatment produced only modest overall effects in this region.

For visualization purposes, panel D displays data collapsed across sex and pretreatment to illustrate the main effect of chronic treatment.

## Discussion

Polysubstance use involving opioids and psychostimulants has become increasingly common and is associated with elevated overdose risk, relapse vulnerability, and reduced treatment efficacy. Despite this growing clinical concern, the neurobehavioral consequences of opioid–stimulant exposure remain poorly understood, particularly with respect to socioaffective dysfunction during withdrawal. In the present study, repeated methamphetamine + fentanyl administration produced a robust reduction in social preference during withdrawal, replicating our previous findings in an independent cohort. Although psilocybin pretreatment administered 24 hours prior to testing did not restore social motivation, it produced drug history-dependent regulation of *CRHR1* expression within the medial prefrontal cortex. In parallel, withdrawal from repeated methamphetamine + fentanyl administration induced pronounced transcriptional adaptations within corticolimbic reward circuitry, including reduced *CRHR1* expression in the NAc and sex-dependent regulation of *OPRM1* expression across cortical and accumbal regions. Together, these findings suggest that opioid–stimulant use produces coordinated alterations in stress and reward signaling pathways that may contribute to withdrawal-associated social dysfunction.

### Repeated methamphetamine + fentanyl polysubstance administration produces persistent social deficits during withdrawal

In this study, we found that withdrawal from repeated methamphetamine + fentanyl treatment induced a significant decline in social preference. Importantly, this behavioral phenotype replicates our previous observations in an independent cohort, strengthening the reproducibility of polysubstance-induced social deficits and supporting social withdrawal as a translationally relevant endpoint in polysubstance models^13^. Social interaction is a critical component of motivational regulation and is increasingly recognized as a protective factor against relapse^52,53^. Conversely, withdrawal-associated reductions in social motivation may contribute to negative affective states and heightened vulnerability to drug-seeking. The emergence of persistent social deficits following combined methamphetamine + fentanyl treatment further suggests that polysubstance use may engage neuroadaptations distinct from those produced by either drug alone.

### Psilocybin did not reverse polysubstance-induced sociability deficits at the assessed timepoint

Despite our previous finding that the 5-HT_2A_R agonist DOI reverses polysubstance withdrawal-induced social deficits^13^, psilocybin treatment did not rescue polysubstance-induced social deficits nor did it alter social preference in saline-treated controls. However, DOI was administered shortly before social testing and can exert immediate behavioral modulation through rapid serotonergic engagement of cortical circuitry^20,54^. In contrast, psilocybin was given 24-hours prior to testing, at a timepoint that engages sustained neuroplasticity-related mechanisms within cortical circuits^21-23^. Thus, future studies will be necessary to determine whether active serotonergic stimulation during sociability is necessary to restore social deficits or whether behavioral recovery by psilocybin may require different post-treatment intervals, repeated dosing paradigms, or additional environmental and experimental factors that interact with psychedelic-induced plasticity.

#### Cortical CRHR1 regulation reflects drug-history-dependent effects of psilocybin

At the molecular level, our findings reveal that psilocybin produced a drug history-dependent regulation of *CHRH1* expression in the medial prefrontal cortex. Specifically, psilocybin decreased *CRHR1* expression in saline-treated control animals but increased *CRHR1* expression following polysubstance treatment. This bidirectional transcriptional response suggests that prior drug history fundamentally alters how cortical stress-signaling pathways respond to psychedelic treatment.

CRH signaling through *CRHR1* is a central component of the brain’s stress-response system and is strongly implicated in the negative emotional states that emerge during drug withdrawal^55^. Increased CRH signaling within corticolimbic circuits has been associated with anxiety-like behavior, dysphoria, and relapse vulnerability across multiple substance use models^40,41^. Within the prefrontal cortex, *CRHR1* signaling contributes to the regulation of attentive processing, stress reactivity, and top-down control over motivational circuitry^56,57^. Consequently, alterations in cortical *CRHR1* expression during withdrawal may influence the integration of stress-related signals that shape the behavioral responses to drug abstinence.

The opposing direction of psilocybin’s transcriptional effects across drug history may reflect state-dependent plasticity within prefrontal cortical networks. Chronic drug exposure is known to remodel transcriptional processes and synaptic architecture within the mPFC, producing lasting alterations in excitatory signaling and stress responsivity^58^. Under these conditions, psychedelic-induced plasticity mechanisms may engage adaptive pathways differently than in drug-naive circuits. Thus, psilocybin may not uniformly suppress stress signaling but rather recalibrate stress-related transcriptional systems depending on the underlying neurobiological state. Future studies examining cell-type specific expression patterns and downstream CRH signaling pathways will be necessary to determine how these transcriptional changes translate into functional circuit adaptations.

#### Polysubstance exposure suppresses CRHR1 expression within the NAc

In contrast to the drug history-dependent effects observed in the mPFC, *CRHR1* expression within the nucleus accumbens was primarily influenced by drug treatment history. Across sexes and pretreatment conditions, polysubstance administration produced a robust reduction in *CRHR1* expression relative to saline controls. These findings suggest that opioid-stimulant use induces transcriptional adaptations within accumbal stress signaling pathways during withdrawal.

The NAc plays a central role in reward valuation and motivational drive, integrating dopaminergic input from the ventral tegmental area with glutamatergic projections from cortical and limbic structures. Emerging evidence indicates that *CRHR1* within the NAc contributes to stress-related modulation of reward processing and drug-seeking behavior^59-61^. Downregulation within the NAc following repeated polysubstance treatment may therefore reflect adaptive responses within reward circuitry as animals transition from drug reinforcement to withdrawal-associated motivational states.

Notably, these reductions in accumbal *CRHR1* occurred independently of psilocybin pretreatment, suggesting that psilocybin’s effects may be more prominent within cortical regulatory circuits than within subcortical reward structures. This dissociation further supports the idea that psychedelic compounds preferentially engage plasticity-related mechanisms within higher-order cortical networks that exert top-down modulation over motivational systems.

### Sex-dependent regulation of μ-opioid receptor expression following polysubstance exposure

In contrast to *CRHR1*, transcriptional regulation of *OPRM1* displayed prominent sex-dependent effects across corticolimbic regions. Within the prefrontal cortex, *OPRM1* expression exhibited a significant main effect of sex and a trend-level Sex x Chronic Treatment interaction. Planned comparisons suggested that polysubstance administration preferentially reduces *OPRM1* expression in males, although this effect did not remain statistically significant after correction for multiple comparisons. Nevertheless, this pattern raises the possibility that cortical μ-opioid signaling may be differentially sensitive to chronic drug exposure in males and females, a hypothesis that warrants further investigation in larger cohorts.

Within the nucleus accumbens, a clear sex-dependent effect emerged. Polysubstance administration produced a pronounced reduction in *OPRM1* expression in females, whereas males exhibited relatively stable expression levels. Moreover, females given repeated methamphetamine + fentanyl displayed significantly lower accumbal *OPRM1* expression compared with males within the same treatment condition.

The μ-opioid receptor plays a fundamental role in mediating reward valuation, social bonding, and affective regulation^39,46^. Endogenous opioid signaling within the mesocorticolimbic system contributes to the reinforcing properties of both natural rewards and psychoactive drugs, while also modulating social behavior^46,47^. Reduced *OPRM1* expression within the NAc may therefore reflect diminished opioid-mediated reward signaling during withdrawal which could contribute to the emergence of negative motivational states.

The pronounced reduction in accumbal *OPRM1* expression observed in females following polysubstance administration suggests that opioid-stimulant combinations can engage sex-specific adaptations within reward circuitry. Sex differences in opioid signaling and dopamine-opioid interactions have been documented across multiple addiction models, and hormonal modulation of opioid receptor expression may further contribute to these divergent responses^62-66^. Thus, the present findings highlight the importance of considering sex as a biological variable when evaluating molecular adaptations to polysubstance exposure.

In addition to these sex-dependent effects, the omnibus model also detected a modest Pretreatment × Chronic Treatment interaction for accumbal *OPRM1* expression, suggesting that the transcriptional impact of psilocybin varies depending on drug history. However, follow-up simple-effects comparisons did not remain statistically significant after correction for multiple testing, indicating that these effects should be interpreted cautiously. Together with the modest main effect of psilocybin pretreatment observed for *CRHR1* expression in the NAc, these findings suggest that psilocybin exerts subtle modulatory influences on accumbal stress- and opioid-related signaling pathways. Nevertheless, these effects were relatively small compared to the robust treatment- and sex-dependent adaptations observed within this region.

### Integrating stress and reward signaling in polysubstance withdrawal

Taken together, the present findings suggest that withdrawal from methamphetamine + fentanyl use produces coordinated transcriptional adaptations across corticolimbic circuits that regulate stress and reward processing. Reductions in accumbal *CRHR1* expression and sex-dependent changes in *OPRM1* expression may reflect alterations in motivational circuitry that contribute to withdrawal-associated social dysfunction. At the same time, psilocybin’s bidirectional regulation of *CRHR1* within the mPFC indicates that psychedelic compounds can engage stress-related transcriptional systems in a drug-history dependent manner.

Although psilocybin did not rescue behavioral social deficits under the present experimental conditions, the observed molecular changes suggest that psychedelic-induced plasticity can still influence circuit-level processes relevant to addiction. These findings underscore the complexity of psychedelic modulation of corticolimbic signaling and highlight the importance of considering drug history, timing of administration, and sex-specific neurobiological factors when evaluating potential therapeutic mechanisms.

Several limitations should be considered when interpreting the present findings. First, gene expression was measured at a single post-withdrawal time point, which limits the ability to determine the temporal dynamics of both withdrawal- and psilocybin-related transcriptional changes. In addition, because tissue was collected only after the withdrawal period, the observed molecular adaptations cannot be definitively attributed to withdrawal per se and may instead reflect persistent consequences of polysubstance treatment, withdrawal-related processes, or an interaction between the two. Future studies incorporating tissue collection both immediately following polysubstance treatment and at multiple withdrawal time points will be important for disentangling the direct effects of repeated methamphetamine-fentanyl administration from neuroadaptations that emerge specifically during withdrawal. Second, the present analysis focused on bulk tissue expression within the mPFC and NAc, and future studies incorporating cell-type specific approaches may help clarify the circuit-level mechanisms underlying these effects. Additionally, behavioral assessments were conducted at a single withdrawal time point following psilocybin administration. Given emerging evidence that psychedelic-induced plasticity evolves over time, future work examining extended post-treatment intervals may reveal behavioral and neurobiological effects not captured in the present study.

## Conclusion

In summary, repeated methamphetamine-fentanyl administration produces robust social deficits during withdrawal and induces transcriptional adaptations within stress- and reward-related signaling pathways across corticolimbic circuits. Psilocybin pretreatment did not restore social motivation at the time point examined but produced drug-history–dependent regulation of cortical *CRHR1* expression. In parallel, withdrawal from repeated polysubstance administration induced region- and sex-specific alterations in μ-opioid receptor expression within reward circuitry. Together, these findings, which are summarized in Table 1, highlight complex interactions between stress signaling, opioid receptor regulation, and psychedelic-induced plasticity in the context of polysubstance withdrawal and provide new insights into the molecular substrates underlying socioaffective dysfunction in addiction.

## Figures and Tables

**Table 1:** Summary of region-specific transcriptional adaptations following polysubstance and psilocybin pretreatment. Key statistical findings for *OPRM1* and *CRHR1* expression in the medial prefrontal cortex (mPFC) and nucleus accumbens (NAc), highlighting sex-dependent opioid receptor regulation and drug-history–dependent modulation of cortical *CRHR1* signaling

Brain Region	Gene	Key Statistical Effect	Interpretation
mPFC	*OPRM1*	Main Effect of Sex	Sex differences in cortical *OPRM1* transcription with possible sex-dependent drug regulation
NAc	*OPRM1*	Sex x Chronic Treatment Interaction	Females show strong reductions in *OPRM1* expression following polysubstance exposure
mPFC	*CRHR*1	Pretreatment x Chronic Treatment Interaction	Psilocybin bidirectionally regulates *CRHR1* expression depending on prior polysubstance exposure
NAc	*CRHR1*	Main Effect of Chronic Treatment	Polysubstance exposure suppresses *CRHR1* expression

## Data Availability

All data supporting the findings of this study are available upon request to the authors.

## References

[1] Crummy EA, O’Neal TJ, Baskin BM, Ferguson SM (2020) One Is Not Enough: Understanding and Modeling Polysubstance Use. Front Neurosci 14:569 32612502 10.3389/fnins.2020.00569 PMC7309369

[2] Friedman J, Shover CL (2023) Charting the fourth wave: Geographic, temporal, race/ethnicity and demographic trends in polysubstance fentanyl overdose deaths in the United States, 2010–2021. Addict Abingdon Engl 118:2477–2485 10.1111/add.16318 37705148

[3] Rockhill K, Black J, Iwanicki J, Abraham A (2025) Polysubstance Use Profiles Among the General Adult Population, United States, 2022. Am J Public Health 115:747–757 40112266 10.2105/AJPH.2024.307979 PMC11983067

[4] Connor JP, Gullo MJ, White A, Kelly AB (2014) Polysubstance use: diagnostic challenges, patterns of use and health. Curr Opin Psychiatry 27:269–275 24852056 10.1097/YCO.0000000000000069

[5] Strickland JC, Smith MA (2014) The Effects of Social Contact on Drug Use: Behavioral Mechanisms Controlling Drug Intake. Exp Clin Psychopharmacol 22:23–34 24188170 10.1037/a0034669 PMC3926100

[6] Christie NC (2021) The role of social isolation in opioid addiction. Soc Cogn Affect Neurosci 16:645–656 33681992 10.1093/scan/nsab029 PMC8259283

[7] McDonagh J, Williams CB, Oldfield BJ, Cruz-Jose D, Olson DP (2020) The Association of Loneliness and Non-prescribed Opioid Use in Patients With Opioid Use Disorder. J Addict Med 14:489–493 32039936 10.1097/ADM.0000000000000629

[8] McDonald S, Darke S, Kaye S, Torok M (2013) Deficits in social perception in opioid maintenance patients, abstinent opioid users and non-opioid users. Addict Abingdon Engl 108:566–574 10.1111/add.12040 23164063

[9] Galiza Soares JA, Sutley-Koury SN, Pomrenze MB, Tucciarone JM (2024) Opioidergic tuning of social attachment: reciprocal relationship between social deprivation and opioid abuse. Front Neuroanat 18:1521016 39917739 10.3389/fnana.2024.1521016 PMC11798945

[10] Pomrenze MB (2022) Modulation of 5-HT release by dynorphin mediates social deficits during opioid withdrawal. Neuron 110:4125–4143e6 36202097 10.1016/j.neuron.2022.09.024 PMC9789200

[11] Quednow BB (2017) Social cognition and interaction in stimulant use disorders. Curr Opin Behav Sci 13:55–62

[12] Ehlert A (2024) Substance Use–Related Alterations of Social Decision Making in a Longitudinal Cohort of Young Adults. Biol Psychiatry Cogn Neurosci Neuroimaging 9:1058–1065 39009135 10.1016/j.bpsc.2024.06.014

[13] Salinsky LM, Diaz KC, Fox JL, Panh SM, Ferguson SM, Fentanyl (2026) Methamphetamine and Polysubstance Use Differentially Affect Locomotor Sensitisation and Social Behaviour in Rats: Psychedelic Treatment Reverses Social Deficits. Addict Biol 31:e70132 41787845 10.1111/adb.70132 PMC12963791

[14] Salinsky LM (2023) μ-opioid receptor agonists and psychedelics: pharmacological opportunities and challenges. Front Pharmacol 14:1239159 37886127 10.3389/fphar.2023.1239159 PMC10598667

[15] Krupp KT (2024) Single administration of a psychedelic [(R)-DOI] influences coping strategies to an escapable social stress. Neuropharmacology 252:109949 38636726 10.1016/j.neuropharm.2024.109949 PMC11073902

[16] McClure-Begley TD, Roth BL (2022) The promises and perils of psychedelic pharmacology for psychiatry. Nat Rev Drug Discov 21:463–473 35301459 10.1038/s41573-022-00421-7

[17] Alexander L (2024) Preclinical models for evaluating psychedelics in the treatment of major depressive disorder. Br J Pharmacol n/a10.1111/bph.17370 PMC7619394 39467003

[18] Cappendijk SLT, Fekkes D, Dzoljic MR (1994) The inhibitory effect of norharman on morphine withdrawal syndrome in rats: Comparison with ibogaine. Behav Brain Res 65:117–119 7880450 10.1016/0166-4328(94)90080-9

[19] Glick SD, Rossman K, Rao NC, Maisonneuve IM, Carlson J (1992) N. Effects of ibogaine on acute signs of morphine withdrawal in rats: Independence from tremor. Neuropharmacology 31:497–500 1528400 10.1016/0028-3908(92)90089-8

[20] Glennon RA, Dukat M (2024) 1-(2,5-Dimethoxy-4-iodophenyl)-2-aminopropane (DOI): From an Obscure to Pivotal Member of the DOX Family of Serotonergic Psychedelic Agents – A Review. ACS Pharmacol Transl Sci 7:1722–1745 38898956 10.1021/acsptsci.4c00157 PMC11184610

[21] Jefsen OH, Elfving B, Wegener G, Müller HK (2021) Transcriptional regulation in the rat prefrontal cortex and hippocampus after a single administration of psilocybin. J Psychopharmacol (Oxf) 35:483–493 10.1177/0269881120959614 33143539

[22] Zhao X (2024) Psilocybin promotes neuroplasticity and induces rapid and sustained antidepressant-like effects in mice. J Psychopharmacol (Oxf) 38:489–499 10.1177/02698811241249436 38680011

[23] Shao L-X (2021) Psilocybin induces rapid and persistent growth of dendritic spines in frontal cortex in vivo. Neuron 109:2535–2544e4 34228959 10.1016/j.neuron.2021.06.008 PMC8376772

[24] The Neuroscience of Drug Reward and Addiction ∣ Physiological Reviews ∣ American Physiological Society Physiol Rev. https://journals.physiology.org/doi/10.1152/physrev.00014.2018 10.1152/physrev.00014.2018 PMC6890985 31507244

[25] Chen L, Kreko-Pierce T, Cassoday SL, Al-Harthi L, Hu X-T (2025) Methamphetamine self-administration causes neuronal dysfunction in rat medial prefrontal cortex in a sex-specific and withdrawal time-dependent manner. Front Pharmacol 16:1527795 40028159 10.3389/fphar.2025.1527795 PMC11868113

[26] Fox ME (2023) Adaptations in nucleus accumbens neuron subtypes mediate negative affective behaviors in fentanyl abstinence. Biol Psychiatry 93:489–501 36435669 10.1016/j.biopsych.2022.08.023 PMC9931633

[27] Wongveerakul P, Cheaha D, Kumarnsit E, Samerphob N (2025) Circuit-specific neural perturbations and recovery in methamphetamine addiction in a mouse model. Neurosci Lett 853:138201 40101836 10.1016/j.neulet.2025.138201

[28] Dawes MH, Ortelli OA, Holleran KM, Jones SR (2024) Fentanyl self-administration is accelerated by methamphetamine co-use and results in worsened hypodopaminergia in male, but not female rats. Eur J Neurosci 60:5912–5926 39251212 10.1111/ejn.16533 PMC11484618

[29] Cole RH, Moussawi K, Joffe ME (2024) Opioid Modulation of Prefrontal Cortex Cells and Circuits. Neuropharmacology 248:109891 38417545 10.1016/j.neuropharm.2024.109891 PMC10939756

[30] Isaac J, Murugan M (2024) Interconnected neural circuits mediating social reward. Trends Neurosci 47:1041–1054 39532581 10.1016/j.tins.2024.10.004 PMC11633286

[31] Isaac J (2024) Sex differences in neural representations of social and nonsocial reward in the medial prefrontal cortex. Nat Commun 15:8018 39271723 10.1038/s41467-024-52294-6 PMC11399386

[32] Dai B (2022) Responses and functions of dopamine in nucleus accumbens core during social behaviors. Cell Rep 40:111246 36001967 10.1016/j.celrep.2022.111246 PMC9511885

[33] Kalivas PW, Volkow ND (2005) The neural basis of addiction: a pathology of motivation and choice. Am J Psychiatry 162:1403–1413 16055761 10.1176/appi.ajp.162.8.1403

[34] Kim Y (2015) Mapping social behavior-induced brain activation at cellular resolution in the mouse. Cell Rep 10:292–305 25558063 10.1016/j.celrep.2014.12.014 PMC4294964

[35] Koob GF, Volkow ND (2016) Neurobiology of addiction: a neurocircuitry analysis. Lancet Psychiatry 3:760–773 27475769 10.1016/S2215-0366(16)00104-8 PMC6135092

[36] Murugan M (2017) Combined Social and Spatial Coding in a Descending Projection from the Prefrontal Cortex. Cell 171:1663–1677e16 29224779 10.1016/j.cell.2017.11.002 PMC5889923

[37] Klawonn AM, Malenka RC (2018) Nucleus Accumbens Modulation in Reward and Aversion. Cold Spring Harb. Symp. Quant. Biol. 83, 119–129 30674650 10.1101/sqb.2018.83.037457 PMC6650377

[38] Volkow ND, Wise RA, Baler R (2017) The dopamine motive system: implications for drug and food addiction. Nat Rev Neurosci 18:741–752 29142296 10.1038/nrn.2017.130

[39] Trezza V, Damsteegt R, Achterberg EJM, Vanderschuren LJ (2011) M. J. Nucleus Accumbens μ-Opioid Receptors Mediate Social Reward. J Neurosci 31:6362–6370 21525276 10.1523/JNEUROSCI.5492-10.2011 PMC3098965

[40] Mantsch JR (2022) Corticotropin releasing factor and drug seeking in substance use disorders: Preclinical evidence and translational limitations. Addict Neurosci 4:100038 36531188 10.1016/j.addicn.2022.100038 PMC9757758

[41] Zorrilla EP, Logrip ML, Koob GF (2014) Corticotropin Releasing Factor: A Key Role in the Neurobiology of Addiction. Front Neuroendocrinol 35:234–244 24456850 10.1016/j.yfrne.2014.01.001 PMC4213066

[42] Mantsch JR (2022) Corticotropin releasing factor and drug seeking in substance use disorders: Preclinical evidence and translational limitations. Addict Neurosci 4:100038 36531188 10.1016/j.addicn.2022.100038 PMC9757758

[43] Daiwile AP, Jayanthi S, Cadet JL (2021) Sex- and Brain Region-specific Changes in Gene Expression in Male and Female Rats as Consequences of Methamphetamine Self-administration and Abstinence. Neuroscience 452:265–279 33242543 10.1016/j.neuroscience.2020.11.025 PMC8175033

[44] Schneider P (2025) Region-specific neuroadaptations of CRF1 and CRF2 expression following heroin exposure in female rats. Pharmacol Biochem Behav 247:173931 39626795 10.1016/j.pbb.2024.173931 PMC11769769

[45] de la Tremblaye PB, Linares NN, Schock S, Plamondon H (2016) Activation of CRHR1 receptors regulates social and depressive-like behaviors and expression of BDNF and TrkB in mesocorticolimbic regions following global cerebral ischemia. Exp Neurol 284:84–97 27498336 10.1016/j.expneurol.2016.07.019

[46] Smith ACW (2024) A master regulator of opioid reward in ventral prefrontal cortex. Science 384:eadn0886 38843332 10.1126/science.adn0886 PMC11323237

[47] Meier IM, van Honk J, Bos PA, Terburg D (2021) A mu-opioid feedback model of human social behavior. Neurosci Biobehav Rev 121:250–258 33359094 10.1016/j.neubiorev.2020.12.013

[48] Morison SL (2025) Recruitment of specific dopamine neuron sub-circuits by opioids. Addict Neurosci 17:100233 41497365 10.1016/j.addicn.2025.100233 PMC12768494

[49] Nichols DE, Psychedelics (2016) Pharmacol Rev 68:264–355 26841800 10.1124/pr.115.011478 PMC4813425

[50] Mathis A (2018) DeepLabCut: markerless pose estimation of user-defined body parts with deep learning. Nat Neurosci 21:1281–1289 30127430 10.1038/s41593-018-0209-y

[51] Goodwin NL (2024) Simple Behavioral Analysis (SimBA) as a platform for explainable machine learning in behavioral neuroscience. Nat Neurosci 27:1411–1424 38778146 10.1038/s41593-024-01649-9 PMC11268425

[52] Venniro M (2018) Volitional social interaction prevents drug addiction in rat models. Nat Neurosci 21:1520–1529 30323276 10.1038/s41593-018-0246-6 PMC7386559

[53] Venniro M (2022) The Protective Effect of Social Reward on Opioid and Psychostimulant Reward and Relapse: Behavior, Pharmacology, and Brain Regions. J Neurosci Off J Soc Neurosci 42:9298–9314 10.1523/JNEUROSCI.0931-22.2022 PMC9794371 36517252

[54] Cameron LP, Jaster AM, Ramos RA, Ullman EZ (2026) The utility of 2,5-dimethoxy-4-iodoamphetamine for the study of serotonin 2A and 2C receptors. Mol Pharmacol 108 10.1016/j.molpha.2025.100093 41447818

[55] Haass-Koffler CL, Bartlett SE (2012) Stress and addiction: contribution of the corticotropin releasing factor (CRF) system in neuroplasticity. Front Mol Neurosci 5 10.3389/fnmol.2012.00091 PMC3434418 22973190

[56] Uribe-Mariño A (2016) Prefrontal Cortex Corticotropin-Releasing Factor Receptor 1 Conveys Acute Stress-Induced Executive Dysfunction. Biol Psychiatry 80:743–753 27318500 10.1016/j.biopsych.2016.03.2106

[57] Hupalo S (2019) Corticotropin-Releasing Factor (CRF) circuit modulation of cognition and motivation. Neurosci Biobehav Rev 103:50–59 31212019 10.1016/j.neubiorev.2019.06.010 PMC6692202

[58] Hearing M (2019) Prefrontal-accumbens opioid plasticity: Implications for relapse and dependence. Pharmacol Res 139:158–165 30465850 10.1016/j.phrs.2018.11.012 PMC6360131

[59] Lemos JC (2012) Severe stress switches CRF action in the nucleus accumbens from appetitive to aversive. Nature 490:402–406 22992525 10.1038/nature11436 PMC3475726

[60] Uribe KP (2020) Overexpression of corticotropin-releasing factor in the nucleus accumbens enhances the reinforcing effects of nicotine in intact female versus male and ovariectomized female rats. Neuropsychopharmacology 45:394–403 31614362 10.1038/s41386-019-0543-0 PMC6901467

[61] Borrego MB (2022) Central nucleus of the amygdala projections onto the nucleus accumbens core regulate binge-like alcohol drinking in a CRF-dependent manner. Neuropharmacology 203:108874 34748860 10.1016/j.neuropharm.2021.108874 PMC10578155

[62] Nicolas C (2021) Sex differences in opioid and psychostimulant craving and relapse: a critical review. 03.30.21254644 Preprint at 10.1101/2021.03.30.21254644 (2021) PMC11060335 34987089

[63] Zamfir M (2023) Distinct and sex-specific expression of mu opioid receptors in anterior cingulate and somatosensory S1 cortical areas. Pain 164:703 35973045 10.1097/j.pain.0000000000002751 PMC10026835

[64] Teodorov E, Camarini R, Bernardi MM, Felicio LF (2014) Treatment with steroid hormones and morphine alters general activity, sexual behavior, and opioid gene expression in female rats. Life Sci 104:47–54 24699004 10.1016/j.lfs.2014.03.021

[65] Mirbaha H, Tabaeizadeh M, Shaterian-Mohammadi H, Tahsili-Fahadan P, Dehpour AR (2009) Estrogen pretreatment modulates morphine-induced conditioned place preference in ovariectomized mice. Pharmacol Biochem Behav 92:399–403 19463255 10.1016/j.pbb.2009.01.009

[66] Ethridge SB, Smith MA (2023) Estradiol and Mu opioid-mediated reward: The role of estrogen receptors in opioid use. Addict Neurosci 9:100139 38155959 10.1016/j.addicn.2023.100139 PMC10753849

